# Significant Improvement in Domestic Violence and Aggression in comorbid ADHD and Conduct Disorder Following Methylphenidate Initiation: A Case Report

**DOI:** 10.1192/bjo.2026.11799

**Published:** 2026-06-30

**Authors:** Zaid Mamser

**Affiliations:** Lancashire and South Cumbria Foundation Trust, Preston, United Kingdom

## Abstract

**Aims:**

Attention Deficit Hyperactivity Disorder (ADHD) and Conduct Disorder (CD) are diagnoses that are frequently comorbid; the DSM-5-TR notes that 25% of patients with ADHD combined type present with CD. CD is characterized by persistent dissocial, aggressive, and defiant behaviors that defy age appropriate expectations. NICE guidelines and literature suggest that psychostimulants, specifically Methylphenidate, are indicated in cases where ADHD and CD arecomorbid.

**Methods:**

This is the case of a 15-year-old male adoptee referred to Tier 3 CAMHS for severe aggression, impulsivity, and domestic violence. The patient exhibited coercive control toward his mother, physical violence involving weapons, and property damage, necessitating police involvement and risking family placement breakdown.

Following a failure of psychosocial interventions, the patient underwent a Qb Test and Conners 4 assessments and review by a consultant psychiatrist confirming ADHD and Conduct Disorder Diagnosis. Methylphenidate (Concerta XL) was initiated and titrated to 36mg daily.

Outcomes were significant. Within one month, antisocial behaviors reduced markedly. At seven months, domestic violence had ceased, and social care involvement ended. The patient successfully completed his GCSEs. Repeat testing on medication showed normalisation of scores: Qb Test activity dropped to the 23rd percentile and impulsivity to the 21st percentile. on repeat parent-rated Conners 4 scores for Conduct Disorder dropped to zero.

**Results:**

This case highlights the importance of identifying and treating underlying ADHD in adolescents presenting with severe conduct issues. Higher doses of Methylphenidate are often cited as beneficial for CD symptom reduction, this patient achieved remission of aggression at 36mg/day. The case shows that delaying ADHD diagnosis in favor of solely managing behavioral conduct symptoms can risk education and family stability. Treating the ADHD effectively mitigated the emotional dysregulation and violence, and reduced the negative impact on this patient during a crucial period of his life as he was completing high school.

**Conclusion:**

This case highlights the importance of considering comorbid ADHD and Conduct disorder in patients presenting with impulsive behaviours and aggression as their primary presenting complaints. As appropriate management with Methylphenidate could have a significant therapeutic effect on these patients and their antisocial behaviours.

